# Supplementary material for: Integrated Transcriptome and Metabolome Analyses Uncover Cholesterol-Responsive Gene Networks
Source: Int J Mol Sci. 2025 Jul 23;26(15):7108. doi: 10.3390/ijms26157108 (PMC12346660; doi:10.3390/ijms26157108)
Supplement: Supplementary file 1 [file ijms-26-07108-s001.zip › Supplementary Figure.pdf]

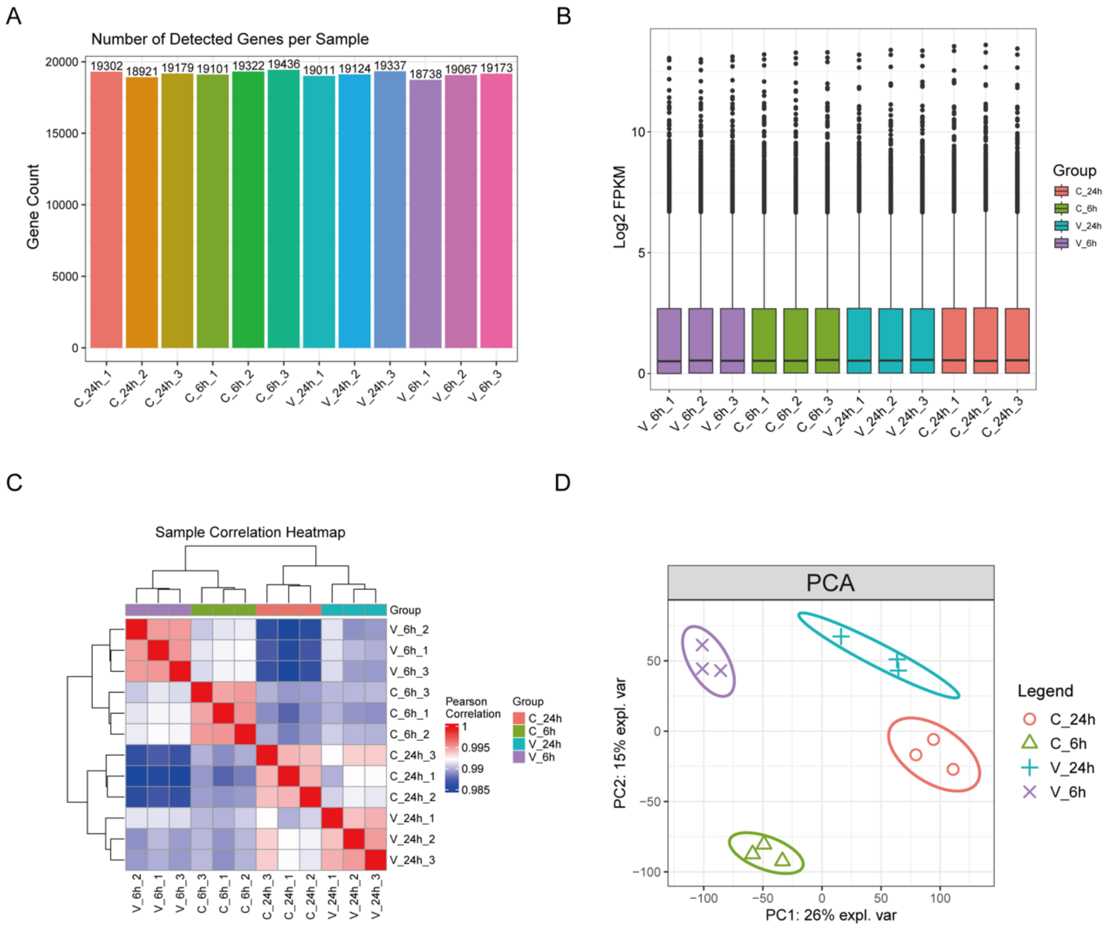

Figure S1. Basic data characteristics and sample clustering analysis. **(A)** Detected gene counts per HeLa sample (12 total), grouped by treatment and time. **(B)** Box - plot of Log2 FPKM across samples. **(C)** Heatmap of sample - to - sample Pearson correlations. **(D)** PCA of samples. Clusters separate by treatment (V/C) and time (6h/24h).

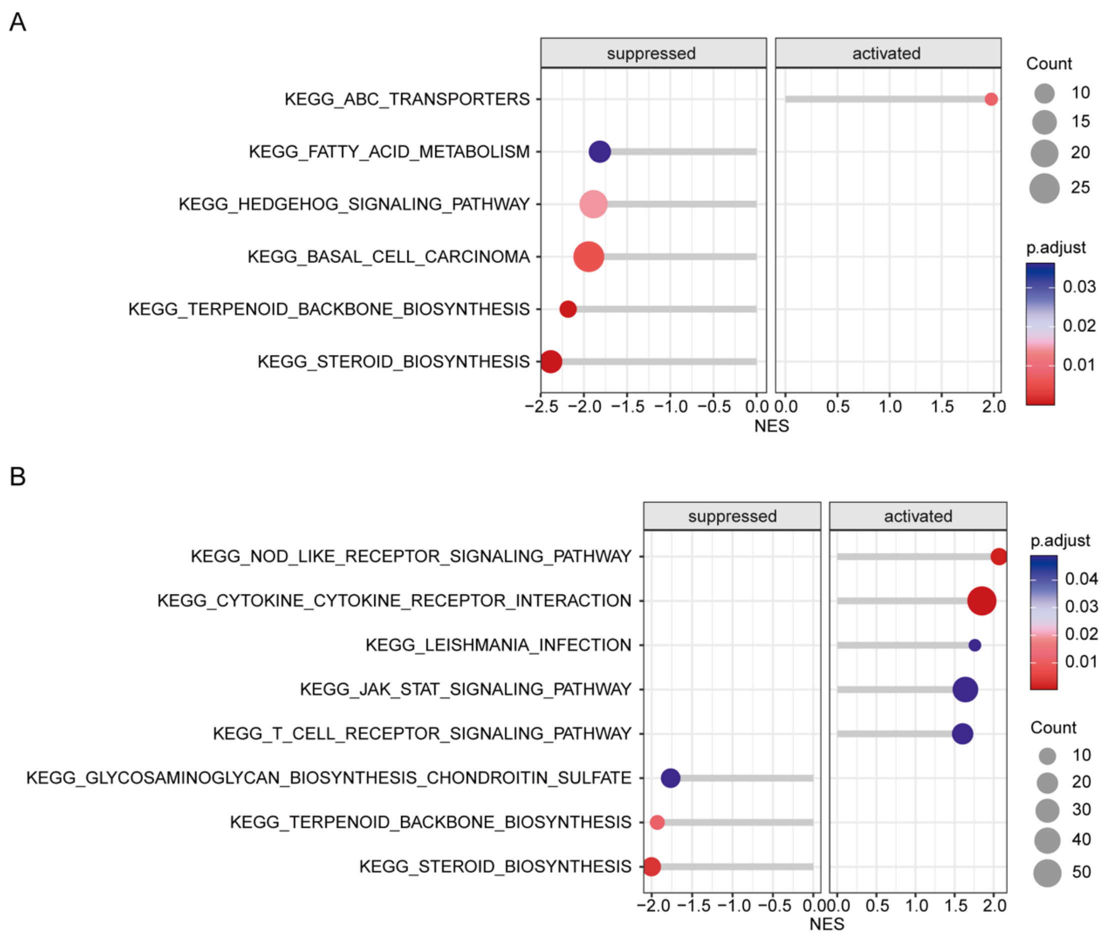

Figure S2. Analysis of the KEGG pathways based on GSEA. **(A)** GSEA-KEGG analysis results for 6 - hour cholesterol - exposed HeLa cells. Pathways are categorized as suppressed (left) or activated (right). Normalized Enrichment Score (NES), adjusted p-value (p.adj), and gene count information are presented, illustrating pathway level responses. **(B)** GSEA-KEGG analysis results for 24 - hour cholesterol exposed HeLa cells. Similar to **(A)**, pathways are shown as suppressed or activated.

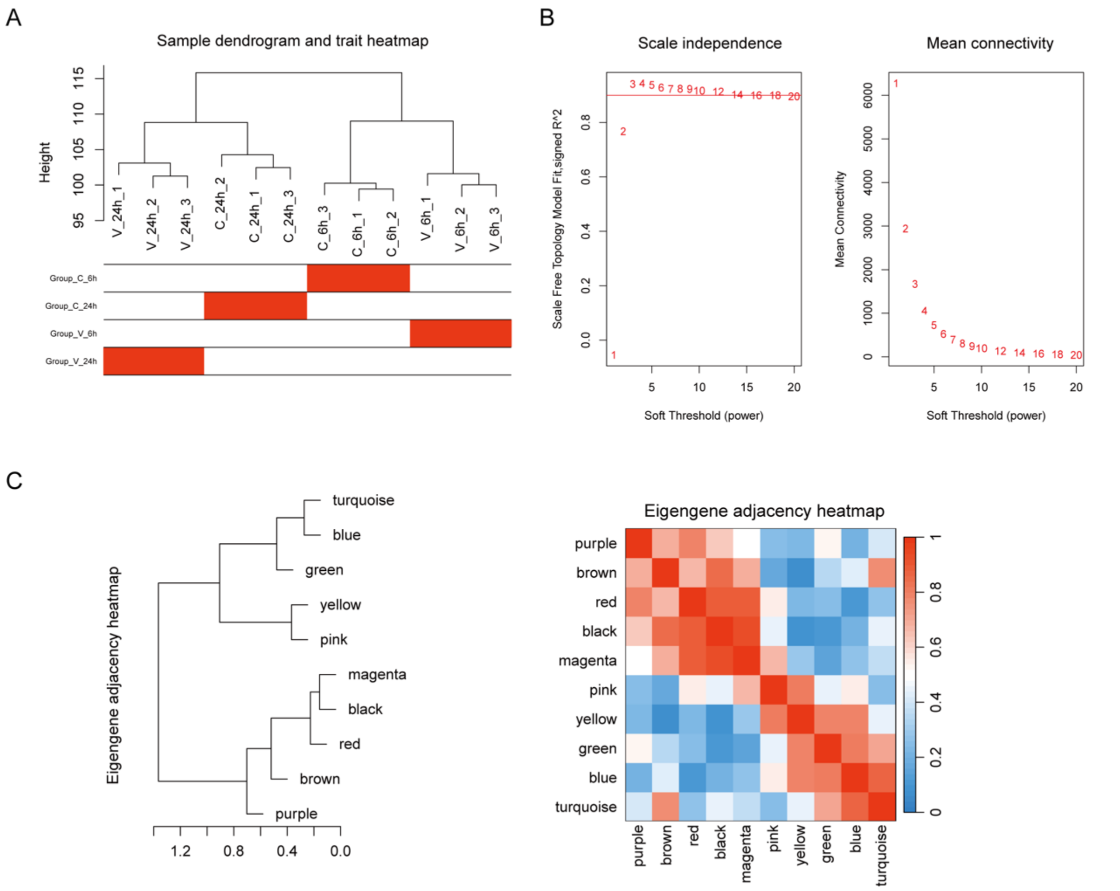

Figure S3. Sample Clustering and Co - expression Network Validation. **(A)** Sample dendrogram and trait heatmap. Dendrogram shows hierarchical clustering of HeLa cell samples (6h/24h, control "V" vs cholesterol - treated "C") with trait heatmap. **(B)** Scale Independence and Mean Connectivity Plots validate scale - free network topology. Left: Scale independence (R^2>0.9 at soft - threshold) confirms suitable power for network construction. Right: Mean connectivity curves guide threshold selection, ensuring network adherence to scale - free properties. **(C)** Eigengene adjacency heatmap. Heatmap visualizes co - expression relationships among modules (color - coded).

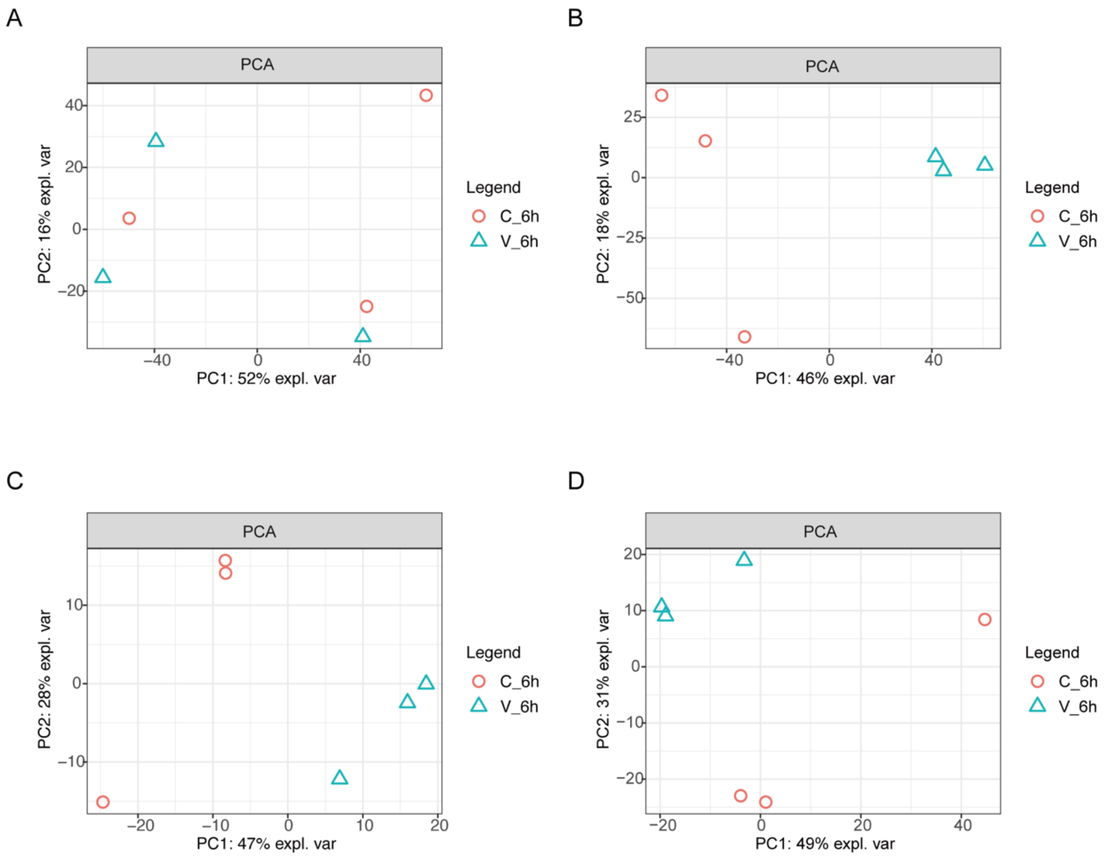

Figure S4. Non-targeted metabolomics PCA analysis. PCA of untargeted metabolomics data from HeLa cells with/without 6h cholesterol treatment. **(A)** negative ion mode, lipidome. **(B)** positive ion mode, lipidome. **(C)** negative ion mode, water - soluble. **(D)** positive ion mode, water - soluble.
